# *Abelmoschus eculentus* Seed Extract Exhibits In Vitro and In Vivo Anti-Alzheimer’s Potential Supported by Metabolomic and Computational Investigation

**DOI:** 10.3390/plants12122382

**Published:** 2023-06-20

**Authors:** Hussain T. Bakhsh, Fatma A. Mokhtar, Abeer H. Elmaidomy, Hanan F. Aly, Eman A. Younis, Mubarak A. Alzubaidi, Faisal H. Altemani, Naseh A. Algehainy, Mohammed Ali A. Majrashi, Faisal Alsenani, Gerhard Bringmann, Usama Ramadan Abdelmohsen, Omnia Hesham Abdelhafez

**Affiliations:** 1 Department of Pharmacy Practice, Faculty of Pharmacy, King Abdulaziz University, Jeddah 21589, Saudi Arabia; htbakhsh@kau.edu.sa; 2 Department of Pharmacognosy, Faculty of Pharmacy, El Saleheya El Gadida University, El Saleheya El Gadida, Sharkia 44813, Egypt; 3 Department of Pharmacognosy, Faculty of Pharmacy, Beni-Suef University, Beni-Suef 62514, Egypt; abeerhelmaidomy@yahoo.com; 4 Department of Therapeutic Chemistry, National Research Centre (NRC), El-Bouth St., Cairo 12622, Egypt; hanan_abduallah@yahoo.com (H.F.A.); youniseman530@yahoo.com (E.A.Y.); 5 Department of Biological Sciences, Faculty of Science, King Abdulaziz University, Jeddah 21589, Saudi Arabia; mahalzubaidi@kau.edu.sa; 6 Department of Medical Laboratory Technology, Faculty of Applied Medical Sciences, University of Tabuk, Tabuk 71491, Saudi Arabia; faltemani@ut.edu.sa (F.H.A.); nalgehainy@ut.edu.sa (N.A.A.); 7 Department of Pharmacology, College of Medicine, University of Jeddah, Jeddah 23890, Saudi Arabia; mamajrashi@uj.edu.sa; 8 Department of Pharmacognosy, College of Pharmacy, Umm Al-Qura University, Makkah 21955, Saudi Arabia; fssenani@uqu.edu.sa; 9 Institute of Organic Chemistry, University of Würzburg, Am Hubland, 97074 Würzburg, Germany; 10 Department of Pharmacognosy, Faculty of Pharmacy, Minia University, Minia 61519, Egypt; 11 Department of Pharmacognosy, Faculty of Pharmacy, Deraya University, 7 Universities Zone, New Minia 61111, Egypt

**Keywords:** Alzheimer’s, acetyl choline, BDNF, seeds, pharmacological network, okra

## Abstract

*Abelmoschus esculentus* Linn. (okra, F. Malvaceae) is a fruit widely consumed all over the world. In our study, the anti-Alzheimer’s potential of *A. esculentus* was evaluated. An in vitro DPPH free radical assay on *A. esculentus* seed’s total extract and AChE inhibition potential screening indicated a significant anti-Alzheimer’s activity of the extract, which was confirmed through an in vivo study in an aluminum-intoxicated rat model. Additionally, in vivo results demonstrated significant improvement in Alzheimer’s rats, which was confirmed by improving T-maze, beam balance tests, lower serum levels of AChE, norepinephrine, glycated end products, IL-6, and MDA. The levels of dopamine, BDNF, GSH, and TAC returned to normal values during the study. Moreover, histological investigations of brain tissue revealed that the destruction in collagen fiber nearly returns back to the normal pattern. Metabolomic analysis of the ethanolic extract of *A. esculentus* seeds via LC–HR-ESI-MS dereplicated ten compounds. A network pharmacology study displayed the relation between identified compounds and 136 genes, among which 84 genes related to Alzheimer’s disorders, and focused on AChE, APP, BACE1, MAPT and TNF genes with interactions to all Alzheimer’s disorders. Consequently, the results revealed in our study grant potential dietary elements for the management of Alzheimer’s disorders.

## 1. Introduction

Continuous damage of neuronal construction or performance are the main features of neurodegenerative diseases [1]. Additionally, they act as a chief socioeconomic burden all over the world. The possibility of obtaining a neurodegenerative disorder rises significantly as age increase [1]. Accordingly, with an aging population, the number of affected people has increased even further, demanding the innovation of therapeutic approaches able to decrease or inhibit neurodegenerative illnesses. In the same context, Alzheimer’s disorder (AD) is a neurodegenerative illness which is characterized by damage in brain neurons, especially in those responsible for memory, language, and thinking. Additionally, numerous neurons are destroyed and more brain parts are influenced, which negatively affects the patient’s life [2]. According to the 2022 report of the World Health Organization, 55 million people were affected by AD up until 2019, which is assumed to increase to 139 million by 2050 [3]. Currently, different drugs with different targets for AD treatment are available on the market, such as donepezil hydrochloride, which is an acetylcholinesterase (AChE) inhibitor mostly utilized for Alzheimer’s disorder treatment. Donepezil is FDA-permitted in patients at different stages of Alzheimer’s disorders. Until now, there has been no proof that donepezil changes the disease’s progression. However, it affects various symptoms by cognition and/or behavior improvement. Moreover, it has different off-label uses. This study will point out the possible mechanism of action, the adverse event outline, the pharmacology, the monitoring, and relevant donepezil interactions, significant for members of the interprofessional side for the treatment of patients with Alzheimer’s disorders. The most frequent adverse effects of the drug are gastrointestinal disorders as nausea, diarrhea, and vomiting. Additional common adverse effects comprise insomnia, muscle cramps, weakness, and anorexia, which are more frequent at higher doses. However, most patients suffer from theses adverse effects in minor and transient form, persisting up to three weeks and mostly getting resolved with continual use [4].

On the other hand, neuronal survival and growth mainly depend on neurotrophins, such as brain-derived neurotrophic factor (BDNF). The signaling pathway presented by BDNF and its receptor are the main synaptic plasticity regulators, which contributes an essential function in learning and memory processes [1]. Additionally, BDNF attracts more attention for the innovation of medicines to cure neurological diseases [1,5]. Moreover, several inflammatory mediators have been included in AD, such as tumor necrosis factor-α (TNF-α) as well as interleukin-6 (IL-6) [6]. Different studies showed that TNF-α provokes diminishing in memory as well as peripheral glucose intolerance by disordering insulin signaling and eliciting cellular stress cascades in AD mouse models [7]. During the initial-phase amyloid plaque construction in AD brains, IL-6 was recognized as a major factor in tau phosphorylation, synapse loss, and learning disorders in mice [7]. Regardless of various arguments in the literature, earlier meta-analyses revealed that the IL-6 levels are raised in both cerebrospinal fluid (CSF) and mild cognitive impairment (MCI) plasma and in patients with AD in comparison to control persons [7]. However, this hints at an involvement of IL-6 in AD disorders, and a fundamental association between IL-6, cognitive impairments, and peripheral metabolic disorganization in AD continues to be recognized.

Additionally, amyloid precursor protein (APP) is affected by *β*- and *γ*-secretase to generate amyloid *β* peptide (Aβ), which disperses in neuronal cells in the form of an amyloid residue. The residue is a pathologic property of AD [5,8]. The major source of neurotoxicity in AD was defined as glyceraldehyde-derived advanced glycated end products (AGEs). APP processing and Aβ production are controlled by oxidative stress [8]. Furthermore, neuronal cell death is caused by Aβ through reactive oxygen species (ROS) [9]. AGEs adjust Aβ accumulation and amyloid aggregation [10]. Our results in a former study recommended that glyceraldehyde-deprived AGEs raise the levels of APP and Aβ via ROS, and that this effect finally precedes to cell death [5,8]. Conversely, AGEs role in the enhancement of Alzheimer’s disorder (AD) still indistinguishable.

Nature as a treasure resource of bioactive metabolites provides us with several nutrients helping in curing and decreasing the symptoms of AD [11]. Several vitamins, such as B_6_, B_9_, K, C, and E help in decreasing oxidative stress and improving brain functions [12]. Additionally, some plant extracts possess potential anti-AD properties, such as *Punica granatum* [13], *Teucrium polium* [14], *Fragaria ananassa* [15], *Bacopa floribunda* [16], and *Ginkgo biloba* [17]. On the other hand, *Abelmoschus esculentus* L., synonym: *Hibiscus esculentus*, mostly identified as okra or lady´s fingers, is a nutrient-rich food. It acts as a major resource of various valuable constituents such as flavonoids, phenolic acids, mucilage, vitamins, nutrients, fibers, and minerals [18]. Different parts of okra, such as fruits, seeds, and leaves, are implicated in various applications owing to their properties and composition [19]. Additionally, it was used over centuries in traditional medicine for the treatment of various illnesses, such as worm infestation, dysentery, and inflammation and irritation of the stomach, intestines, and kidneys. Okra fruit extract, as well as its derived flavonoids and polysaccharides, showed remarkable anti-AD potential [20,21,22,23,24]. However, limited studies have been conducted on okra seeds and their oil, which revealed their polyphenolic compounds and polyunsaturated fatty acids [25,26,27]. A few studies were carried out on the bioactivity of okra seed extract, showing their potent anti-oxidant and anticancer potential against human breast cancer (MCF7) and skin fibroblast (CCD-1059Sk) cells [28]. Consequently, there is a great demand to explore the potential of okra seeds as a low-cost available nutrient.

Aluminum (Al) serves as a crucial risk factor for different age-related neurodegenerative diseases [29], including AD [30]. Aluminum chloride (AlCl_3_) is used as a neurotoxicant, which dispersed in the brain and influences ionic, cholinergic, as well as dopaminergic neurotransmission with negative effect [31]. As a consequence, the aim of our present study was to pinpoint the possible benefits and therapeutic influences of *A. esculentus* seed extract in diminishing the neurodegenerative character of Alzheimer’s disorder in a rat model intoxicated with Al, which may afford an economical, reliable, simple, noninvasive, and reproducible blood-based group of significant biomarkers, to prepare the way for feasible early curative approaches. Moreover, a metabolomics study of the extract and a pharmacological network were applied to identify chemical molecules that assist in anti-Alzheimer’s activity.

## 2. Results

### 2.1. In Vitro DPPH Radical Scavenging Activity Assay of A. esculentus Seed Extract

The free radical scavenging potential of *A. esculentus* seed total extract was investigated using the DPPH method [32]. The results demonstrated that *A. esculentus* seed extract had considerable scavenging activity percentages for DPPH, respectively, in a dose dependent manner compared to ascorbic acid (Table 1).

### 2.2. In Vitro Evaluation of Cholinesterase Potential of the A. esculentus Seed Extract

The cholinesterase inhibitory activity of *A. esculentus* seed extract in comparison with the standard drug donepezil were evaluated against the AChE enzyme and the results were obtained as IC_50_ (μg/mL) measures in Table S1. *A. esculentus* seed extract exhibited a noticeable inhibitory activity with an IC_50_ value of 0.028 μg/mL. The *A. esculentus* seed extract showed an AChE inhibitory effect approximately the same as the reference drug (IC_50_ = 0.025 μg/mL) (Table S1).

### 2.3. Acute Toxicity Study

Different concentrations of *A. esculentus* seed extract were utilized for evaluating the acute toxicity, with 500, 1000, 2000, 3000, 4000, as well as 5000 mg/kg b.wt., with four rats for each group (in total, 24 rats were included for all groups together). Until 3000 mg/kg b.wt. and for 48 h, no mortality in addition no toxicity signals were observed as well as no signs of toxicity or behavior abnormalities. The chosen dose utilized was 500 mg/kg b.wt. [33].

### 2.4. Potential Activities of A. esculentus Seed Extract in the T-Maze Test

*A. esculentus* seed extract was investigated for its anti-AD ability at a dose of 500 mg/kg b.wt., utilizing the T-maze test. In this context, the results showed a noteworthy raise in time (per seconds) received by rats reaching their food in the T-maze for rats with AlCl_3_-neurotoxicant (AD group), indicating a worsened neurocognitive function, with an increase in a percentage to 233.8% (Table S2). The rats of the AD group treated with *A. esculentus* seed extract, on the contrary, exhibited a significant decline in time taken to reach their food in the T-maze, compared to the AD-induced group, showing improved cognitive functions, with the improvement percentage reaching 175.5% (6 weeks treatment), compared to donepezil as the standard drug (181.9%); see Table S2.

### 2.5. Potential Effects of A. esculentus Seed Extract in the Beam Balance Test

The results obtained from the beam balance test revealed that AlCl_3_ caused major worsening in brain cognitive abilities for AlCl_3_-neurotoxicant rats (AD group) (Table S3), with a reduction in percentage down to 70.69%. Additionally, rats treated with donepezil or *A. esculentus* seed extract revealed a dramatic enhancement in behavioral status, characterized by motor coordination and enhanced cognition improvement, with a percentage of improvement reaching 62.05% for *A. esculentus* seed extract in comparison with animals treated with donepezil as the standard drug, which recorded 69.02% in a 6-week treatment).

### 2.6. Potential Effects of A. esculentus Seed Extract in Acetylcholine Esterase Levels in the Brain

Table 2 reveals a considerable raise in the AChE in the serum of AD-induced rats with a percentage increase of 94.8%, while the AChE levels showed an improvement upon the treatment of AD rats with *A. esculentus* seed extract, entailing a percentage improvement of 76.2% compared to the standard drug (83.2%).

### 2.7. Potential Effects of A. esculentus Seed Extract in Norepinephrine, Dopamine, and Serotonin Levels in Brain

AD-induced rats revealed a noteworthy rise in the level of norepinephrine, with an increase to 723.0% in comparison with control rats (Table S4), with a promising reduction of both dopamine and 5-HT of 68.9 and 65.6%, respectively. AD rats treated with *A. esculentus* seed extract showed a significant improvement in neurotransmitters levels, reaching 545.0, 46.2, and 50.8% for norepinephrine, dopamine, and 5-HT, respectively, compared to the standard drug (558.0, 55.2, and 54.8% for norepinephrine, dopamine, and 5-HT, respectively). 

### 2.8. Potential Effects of A. esculentus Seed Extract in the Levels of BDNF, Glycated End Product, and IL-6 in the Brain

Table 3 reveals a significant reduction in BDNF level in brain tissue, by 50.3% compared to the control, while a significant elevation in brain tissue glycated end product and serum IL-6 was observed, with an increase reaching 221.2 and 227.7%, respectively, in comparison to the control rats. AD rats treated with *A. esculentus* seed extract exhibited remarkable improvements in BDNF, glycated end product, and IL-6 with percentages amounting to 26.5, 163.7, and 213.5%, respectively, in comparison to the reference drug, which revealed improvements of 35.2, 219.6, and 229.4% for BDNF, AGEs, and IL-6, respectively (Table 3).

### 2.9. Potential Effects of A. esculentus Seed Extract in TAC, MDA, and GSH Levels in the Brain

A significant reduction in TAC was observed with an amount of 74.1% compared to the control. GSH also showed a significant reduction in brain tissue with 60.9%, while a significant increase in MDA level was recorded, reaching a value of 451.7%. AD rats treated with *A. esculentus* seed extract showed an amelioration in anti-oxidant and oxidative stress levels with a higher degree of improvement, reaching 51.6, 390.4, and 44.9% for TAC, MDA, GSH, respectively, compared to donepezil, which recorded improvements of 52.8, 394.2, and 53.4%, respectively (Table S5).

### 2.10. Results of the Histopathological Investigation

**Figure 1:**
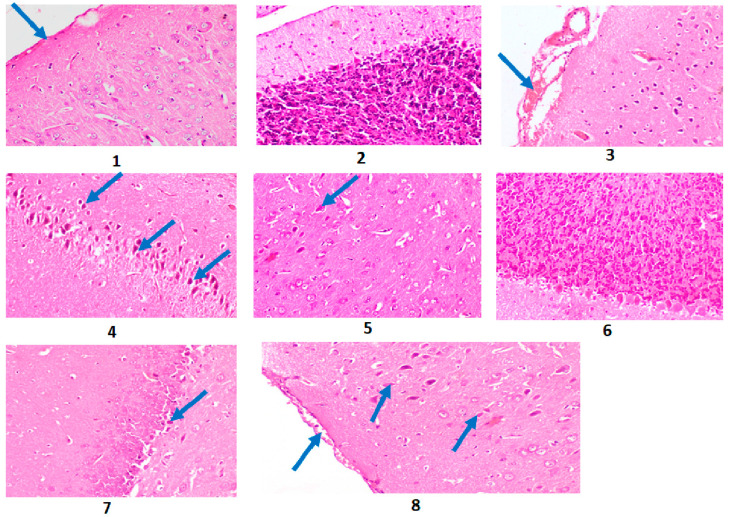
Photomicrographs for H&E-stained sections of the cerebral cortex of rats (scale bar 20 µm): (**1**) Control rat brain revealing normal histological structure of cerebral meninges (arrow). (**2**) Control rat brain displaying the normal histological structure of cerebellum. (**3**) AD rat brain revealing congestion of meningeal blood vessels (arrow) with hemorrhage. (**4**) Alzheimer’s-induced rat brain showing degeneration of hippocampus neurons (arrows). (**5**) AD rat brain treated with *A. esculentus* seeds highlighting a few degenerated neurons in cerebral cortex (arrow). (**6**) AD rat brain treated with *A. esculentus* seeds, showing nearly normal cerebellum. (**7**) AD rat brain treated with *A. esculentus* seeds showing degeneration of few neurons in hippocampus (arrow). (**8**) Donepezil-treated AD rat brain evidencing few degenerated neurons in cerebral cortex, mild meningeal hemorrhage (arrow).

Histopathological investigations showed Alzheimer’s-induced rat brain with the presence of neuronal degeneration and neurofibrillary tangles (NFT), congestion of meningeal blood, degeneration of *Purkinje* cells with decreased granular layer density, and degeneration of hippocampus neurons (Photomicrographs 3 and 4, and Figure 1) compared to control brain rats (Photomicrographs 1 and 2, and Figure 1). After treatment with *A. esculentus*, AD rat brain showed a few degenerated neurons in the cerebral cortex, nearly normal cerebellum, and degeneration of a few neurons in hippocampus (Photomicrographs 5–7, and Figure 1). Donepezil-treated AD rat brain revealed meningeal hemorrhage and a few degenerated neurons in the cerebral cortex (Photomicrograph 8 and Figure 1).

The score order was constructed as follows: score 0 indicates no lesions in all rats of the group (*n* = 5); score 1 indicates (<30%); score 2 indicates (30–50%), score 3 indicates (>50%). Group 1 is the control group; Group 2 is the Alzheimer’s-induced group; Group 3 is the group of AD rats treated with *A. esculentus* seed extract; Group 4 is the donepezil–cured AD group. 

Group 2 (Alzheimer’s-induced rats) showed a high scoring level of histopathological alterations in the brain compared to the control group (Group 1). Group 3 (AD rats treated with *A. esculentus* seed extract) exhibited similar results to Group 4 (donepezil-treated AD rats), in which these groups revealed the same scoring histopathological alterations in brain (Table S6).

### 2.11. Chemical Dereplication of A. esculentus Seed Extract

**Figure 2:**
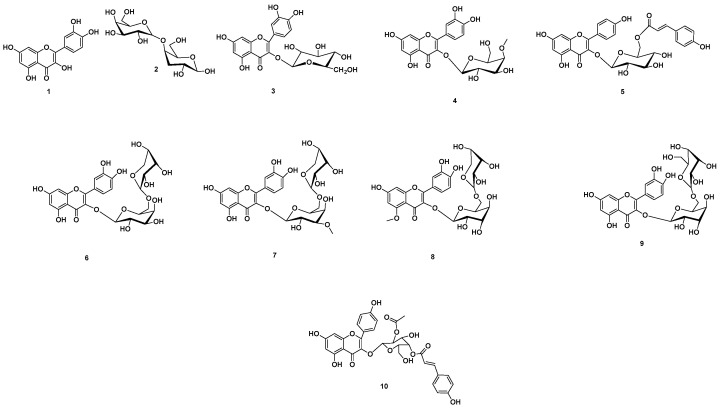
Dereplicated metabolites from LC-HR-ESI-MS analysis of *A. esculentus* seed extract.

Analyzing the total extract of *A. esculentus* seeds, different hits were proposed (Table S7, Figures S1A and S1B, Figure 2). The mass ion peaks at *m*/*z* 301.0353, 341.10792, 465.1033, and 477.10499, in accordance with the suggested molecular formulas C_15_H_10_O_7_, C_12_H_22_O_11,_ C_21_H_20_O_12_, and C_22_H_22_O_12_ [M + H]^+^; [M − H]^+^ (Table S7) fit flavones and disaccharide derivative compounds quercetin (**1**), 4-*O*-*α*-D-galactopyranosyl-D-galactose (**2**), isoquercitrin (**3**), and quercetin 3-glycosides, 3-*O*-(4-*O*-methyl-*β*-D-glucopyranoside) (**4**), which had been previously isolated from *A. manihot* [34], *H. esculentus* [35], and *A. esculentus* [36] (Table S8), respectively.

The molecular ion mass peaks at *m*/*z* 595.1452, 597.1456, 609.1466, 625.1769, 627.1561, and 637.1557 [M + H]^+^; [M - H]^+^ (Table S7), for the predicted molecular formulas C_30_H_26_O_13_, C_26_H_28_O_26_, C_27_H_30_O_16_, C_28_H_32_O_16_, C_27_H_30_O_17_, and C_32_H_28_O_14_, respectively, gave hits of the flavones tiliroside (**5**), 5,7,3′,4′-tetrahydroxy flavonol-3-*O*-[*β*-d-rhamnopyranosil-(1→2)]-*β*-d-glucopyranoside (**6**), quercetin-3-orobinoside (**7**), floramanoside D (**8**), quercetin-3-*O*-sophoroside (**9**), and 3-*O*-kaempferol-2-*O*-acetyl-4-*O*-(*p*-coumaroyl)-α-D-glucopyranoside (**10**), respectively. These compounds had previously been isolated from *A. manihot* [34] and *A. esculentus* [36] (Table S7, Figure 2).

## 3. Discussion

### 3.1. Evaluation of the Anti-Alzheimer’s Potential of A. esculentus Seed Extract

The free radical scavenging potential of *A. esculentus* seed total extract was investigated using the DPPH method [32]. The results demonstrated that *A. esculentus* seed extract provided a considerable scavenging potential for DPPH, in a dose-dependent manner compared to ascorbic acid (Table 1). In addition, the cholinesterase inhibitory activity of *A. esculentus* seed extract in comparison with the standard drug donepezil were evaluated against the AChE enzyme and the results were obtained as IC_50_ values (μg/mL) in Table S1. *A. esculentus* seed extract exhibited a noticeable inhibitory activity with IC_50_ of 0.028 μg/mL. The *A. esculentus* seed extract showed an AChE with approximately the same inhibitory effect as the reference drug (IC_50_ = 0.025 μg/mL) (Table S1).

According to the in vitro DPPH free radical scavenging and the cholinesterase potential assays, *A. esculentus* seed extract was utilized for further in vivo studies for estimating the anti-Alzheimer’s potential.

In accordance with the data represented in Table S2, the results of the behavioral tests approved the earlier obtained ones, which had shown that rats neurointoxicated with AlCl_3_ need additional time to collect food in a T-maze, with an increase of 233.8% compared to control rats, representing a worsened neurocognitive character [37]. *A. esculentus* seed extract, however, exhibited a noteworthy decline in time required by the rats to collect food in the T-maze, in comparison with the AD-induced group. This indicates an improvement in cognitive functions, with improvements reaching 175.5% (6 weeks of treatment) in comparison to the reference drug, which showed an improvement up to 181.9%. A beam balance test is utilized to investigate rodent gait in a testing system which encounters the possibility of them maintaining balance, assumed that the animals need to cross over a raised-up beam with a tight diameter. The findings obtained from the beam balance test showed that AlCl_3_ triggered a major worsening in brain cognitive abilities for rats neurointoxicated with AlCl_3_ (AD group) (Table S3), with a decline of −70.6%. On the other hand, rats treated with donepezil or *A. esculentus* seed extract revealed an improvement in behavioral attitude, which is expressed by values of 69.0 and 62.0%, respectively.

The current results in Table 2 indicate that AChE activity increased significantly in the serum of AlCl_3_-induced AD rats. AlCl_3_ has shown a cholinotoxication that causes changes in different neurotransmission as cholinergic, dopaminergic, and noradrenergic. Accordingly, it is capable of producing weakened cholinergic transmission through influencing the synthesis as well as liberation of neurotransmitters [38]. Our findings are in agreement with previous data reported by Aly et al., 2011 [39], Borai et al., 2017 [37], Aly et al., 2011 [40], and Elmaidomy et al., 2022 [38]. They revealed that AlCl_3_ administration showed a substantial rise in AChE potential in the serum in addition to brain tissue, compared to neurologically normal control rats. Additionally, our results revealed a noteworthy decrease in dopamine (DA) and serotonin, with a significant rise in the norepinephrine level in AD-induced rats. This runs in parallel with Kaur, 2019 [41], who showed the negative effect of AlCl_3_ on memory ability. This was attributed to the involvement of AlCl_3_ in the dopaminergic pathway [38] and also to its function to initiate oxidative stress, as oxidative stress as well as inflammation lead to a decrease in numerous major neurotransmitters, comprising AChE and DA [42]. Likewise, the major change in brain neurotransmitters in rats exposed to AlCl_3_ rats may be attributed to a raised formation of O_2_ and H_2_O_2_, in addition to accumulation of Lewy bodies in the brain, thus raising the probability of neurodegenerative disorders [43]. Moreover, DA oxidation is improved by the elevated concentration of iron in rats exposed to Al, triggering the production of DA quinones, which covalently act together with cysteine deposits of glutathione (GSH) enzymes to inhibit its anti-oxidative potential [43]. Additionally, AlCl_3_ enhances the accumulation of *α*-synuclein and reduces DA-binding receptors (D1 and D2) in the brain cortex in addition to the striatum of AlCl_3_-exposed subjects and in addition to employing an inhibiting potential on different levels, including inhibition of DA *β*-hydroxylase (responsible of conversion of DA into norepinephrine) and inhibition of tryptophan decarboxylase potential (responsible for DA formation) [38]. The diminished level of DA and changed cholinergic function may also be due to elevated monoamine oxidase (MAO) activity, which leads to an increased degradation of serotonin. Additionally, neurotransmission is negatively affected by AlCl_3_, either by direct inhibition of the enzymes included in neurotransmitter synthesis and/or by utilization or influencing the structural characters of synaptic membranes that might influence the release and/or uptake of these molecules [38].

Treatment of AD rats with *A. esculentus* seed extract led to an enhancement in the neurotransmitter levels in AD-induced rats in comparison with untreated AD rats. These effects may be due to an inhibition of different signaling pathways, comprising interference with the IGF-I (insulin-like growth factor-1) mitogenic pathway [44]. Moreover, neurotoxicity and oxidative stress induced by rotenone (a mitochondrial complex I blocker) in mice was affirmed to be diminished by applying an extract of okra seeds [45]. The oral dose of okra seed extract in mice counterbalanced rotenone-induced oxidative deterioration, restored glutathione levels as well as stimulated the antioxidant defense system (glutathione peroxidase, superoxide dismutase). It also has the ability to decrease the activity of rotenone-induced AChE and to recover dopamine in the striatum [45]. Interestingly, extract of okra seeds was effective in restoring mitochondrial complex activities and reserving their redox state. It has been demonstrated that oral administration of okra seeds has a high tendency to provide neuroprotection against neurotoxicants in addition to other neurodegenerative disorders, such as Parkinson’s disease [45]. 

Our present study has revealed the considerable decrease of BDNF in the brains of AD rats (Table 3). Several studies confirmed the present results and the association between Alzheimer’s disorder and BDNF. The decrease in BDNF protein, as well as mRNA levels in the neocortex in addition to the hippocampus of brain tissue, suggested that BDNF has a key role in Alzheimer’s disorder [1,46,47]. This depletion may be explained on the basis of the fact that BDNF is accompanied with Aβ accumulation, tau phosphorylation, in addition to neuroinflammation, and neuronal apoptosis [1]. Aβ-induced downregulation of BDNF is associated with tau; therefore, Alzheimer’s disorder treatments that emphasize only Aβ may not be valuable if the influence of tau pathology on neurotrophic cascades is not reflected [48]. Interestingly, neuronal abnormality, neuronal loss, synaptic degeneration, and behavioral deficits have been proven to be alleviated by BDNF overexpression or gene delivery. Therefore, this indication affording a new opportunity for the treatment of Alzheimer’s disorder using BDNF remedy [1].

Our current results revealed a noteworthy rise in AGEs in brain tissue of AD rats (Table 3). This elevation in AGEs is due to the modification on APP by β- and γ-secretase, which results in deposition of amyloid (plaques) in neuronal cells [8]. Glyceraldehyde-obtained AGEs are known to be a key resource of neurotoxicity in AD. APP and Aβ were elevated by glyceraldehyde-deprived AGEs via ROS. Neurotoxicity has been proven to be enhanced by the combination of AGEs and Aβ [8]. APP expression elevated by tail vein injection of AGEs, which suggested a connection between AGEs in addition to the development of AD [8]. It was verified that AGEs upregulate APP processing protein (BACE and PS1) as well as Sirt1 expression via ROS, with no effect on the expression of antioxidant genes HO-1 and NQO-1. Additionally, AGEs raise GRP78 expression and improve the cell death-connected pathway p53, bcl-2/bax ratio, caspase 3. These results revealed that AGEs worsen the protective effect on neurons influence of Sirt1 which cause neuronal cell death through ER stress [8]. Earlier studies indicated [8,9] that AGEs elevate production of ROS, which triggers downstream cascades associated with APP processing, Aβ production, Sirt1, and GRP78, and this results in the upregulation of a cell death associated pathway. Additionally, this enhances neuronal cell death, and causes the AD development. Earlier studies had indicated that there is a connection between Sirt1 as well as ROS in neuronal cells [47]. Sirt1 provides a neuroprotective importance by hindering the ROS effects [47]. Sirt1 has also been revealed to diminish the Aβ production through α-secretase activation [49]. Consequently, AGEs raise ROS production, which triggers the downstream pathways of Sirt1, GRP78, APP processing, as well as Aβ production. Moreover, the Aβ aggregation in addition to neurofibrillary tangles are improved. Additionally, this eventually leads to the upregulation of the cell death cascade, which improves the neuronal cell death, causing the progress of AD. The deposition of AGEs is considered as a natural process that elevates gradually with aging; abnormal deposition of AGEs, however, could be triggered with illness, dietary habits, and other factors. AGEs accumulate quickly in the body, then pass into the brain, and consequently turn on the mechanism of AGEs effecting. Finally, it has been proven that the level of AGEs is a significant key point for developing AD [49].

Further, IL-6 exhibited significant elevation in serum of AD rats (Table 3). Lyra e Silva et al., 2021 [50], declared that IL-6 showed negative involvement in memory formation, as blocking IL-6 improves long-term potentiation as well as enhances long-term memory in hippocampus-dependent tasks. By the same authors, astrocytes surrounding both parenchymal in addition to vascular amyloid deposits in AD brains and IL-6 immunoreactivity in astrocytes were found, which were positive for Aβ. Moreover, they revealed that AD patients have elevated IL-6 levels in the brain tissue and plasma, and found that plasma IL-6 associated in a positive manner with brain T2 hyperintensities and negatively with cognitive ability. Central administration of Aβ1–42 in mice induced a systemic IL-6 response that is distinguished by elevated plasma and brain levels of this cytokine [51]. Consequently, these findings showed that the brain might be incorporated with the dysregulation in the circulating IL-6, which occurs in AD, and thus could involve the inflammatory reflex triggered by the vagus nerve [52]. AD rats treated with *A. esculentus* seed extract displayed remarkable improvements in BDNF, glycated end product, and IL-6 amounting to 26.5, 163.7, and 213.5%, in comparison with the reference drug, which showed improvements of 35.2, 219.6, and 229.4% for BDNF, AGEs and IL-6, respectively (Table 3).

Our current study revealed a noteworthy reduction in TAC and GSH in AD-induced rats, while major elevation in MDA level was distinguished (Table S5). The chief principles of mitochondrial dysfunction-induced intracellular damage are considered as the disruption in the antioxidant defense mechanism as well as excessive generation of reactive oxygen species (ROS) [39]. These results are in accordance with Aly et al., 2018 [39], who showed that AlCl_3_-associated neurotoxicity leads to an elevation in lipid peroxidation. Additionally, the present study revealed the connection between the rise in MDA in AD rats with the decrease in GSH and TAC, as involved in the elimination of ROS in brain tissue, proposing a pro-oxidant potential of AlCl_3_. Instead, Sumathi et al., 2013 [53] revealed that AlCl_3_ exposition enhances destruction in neuronal lipids related to modifications in the enzymatic antioxidant defense system. Additionally, the current results indicated a substantial decline in GSH level in the brain tissues of rats triggered with AlCl_3_, which attributed to a high level of H_2_O_2_-induced cytotoxicity in brain endothelial cells due to inhibition of glutathione reductase [39]. The significant reduction in brain TAC in AlCl_3_-induced AD rats attributed to long-term exposure to AlCl_3_, causing a rise in lipid peroxidation as well as a reduction and exhaustion of numerous antioxidant enzymes [38]. Additionally, Aly et al., 2022 [39] demonstrated the reduction in TAC in AD-induced rats by the decline in axonal mitochondria transformation, damage of Golgi, and decrease of synaptic vesicles, which leads to the liberation of oxidative molecules such as hydroperoxide, carbonyls, and peroxynitrites, although there is a decline in antioxidant enzymes and glutathione within the neurons. The ameliorative activity of okra seed crude extract mainly depends on the antioxidant metabolites that exhibit their influences by cooperating with free radicals which could additionally destruct essential metabolites in the body [54]. Moreover, the interactions include the accumulation of peroxides, as well as the scavenging of radicals and chelation to metal ions.

The histopathological investigation revealed AD brain with neuronal degeneration and the existence of neurofibrillary tangles (NFT), congestion of meningeal blood, degeneration of *Purkinje* cells with decreased granular layer density and degeneration of hippocampus neurons compared to control brain rats (Figure 1). These results run in parallel with Aşir and Taş, 2022 [55] and Elmaidomy et al., 2022 [38], who revealed that the cerebral cortexes of AD rats showed numerous neuropathic alterations such as neuronal necrosis, as well as neuronophagia, neurofibrillary tangles formation, and focal gliosis. In addition, the hippocampus of the AD rats demonstrated shrunken and necrotized pyramidal neurons accompanied by the formation of neurofibrillary tangles. However, AD-induced rats treated with okra seed extract exhibited normal cerebellum and degeneration of a few neurons in the hippocampus compared to donepezil as the standard drug, which showed meningeal hemorrhage and few degenerated neurons in cerebral cortex. Additionally, a low lesion score was recorded in treatment groups compared to the AD group (Figure 1). Consequently, the current study revealed the therapeutic importance of okra seed extract on inflammatory and neurotransmitters biomarkers.

### 3.2. Metabolomics Profiling of A. esculentus Seed Extract

Additionally, metabolomics profiling of the crude ethanolic extract of *A. esculentus* seeds was performed to explore its chemical diversity, leading to the identification of ten flavonoid glycoside compounds. In this report, the compounds 4-*O*-*α*-D-galactopyranosyl-D-galactose, quercetin 3-glycosides, 3-*O*-(4-*O*-methyl-*β*-D-glucopyranoside), tiliroside, 5,7,3′,4′-tetrahydroxy flavonol-3-*O*-[*β*-d-rhamnopyranosil-(1→2)]-*β*-d-glucopyranoside, quercetin-3-orobinoside, and 3-*O*-kaempferol-2-*O*-acetyl-4-*O*-(*p*-coumaroyl)-α-D-glucopyranoside are detected herein for the first time in okra seed extract.

### 3.3. Pharmacology Networking

#### 3.3.1. Plant-Compounds Network

A simple network, connecting *A. esculentus* seeds to the identified compounds by LC-HRESIMS, was constructed as a first step to build the network pharmacology of *A. esculentus* seeds’ relation to Alzheimer’s disorders.

#### 3.3.2. Compounds-Genes Network

**Figure 3:**
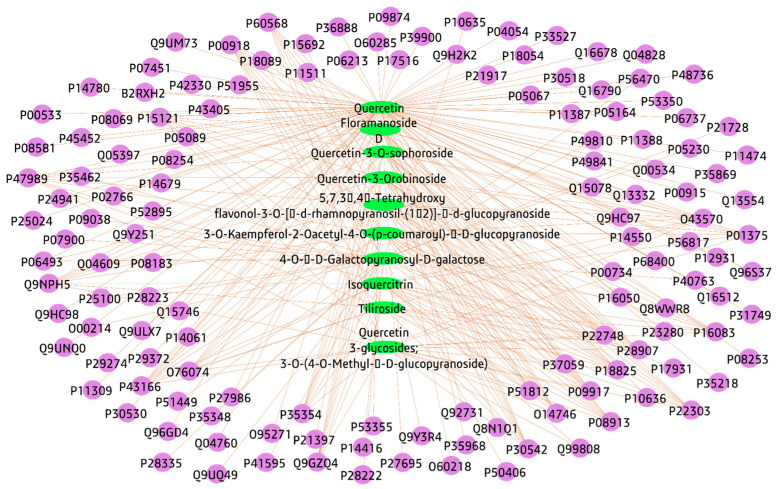
Compounds–genes network, connecting identified compounds from *A. esculentus* seeds to their target genes; oval green shapes represent the identified compounds, the purple circles represent target genes.

The identified compounds targeted genes, these genes were identified by the SwissTargetPrediction database, after omitting the duplicates, a total number of 136 genes were identified and a compounds–genes network was structured and visualized by the Cytoscape software; the network was formed of 146 nodes and 297 edges, with a path length of 2.727 in addition to a network centralization of 0.664 (Figure 3, Table S8). The top gene represented in this network was P00918 with ten edges, followed by eleven genes each with nine edges; Q9NPH5, P15121, P47989, P30542, P43166, O43570, P22748, P22303, P28907, P08913, and P18825 (genes are expressed in UniProt IDs corresponding to gene names CA2, NOX4, AKR1B1, XDH, ADORA1, CA7, CA12, CA4, ACHE, CD38, ADRA2A, and ADRA2C.

#### 3.3.3. Genes–Alzheimer’s Disorders Network

A network linking the identified genes to different Alzheimer’s disorders led to the determination of 84 genes of this data set related to Alzheimer’s disorders; the formed network consisted of 91 nodes and 183 edges with a characteristic path length and network centralization of 2.054 and 0.909, respectively (Figure S2). The formed network identified five genes as having connections to all types of Alzheimer’s disorders; these genes are ACHE, APP, BACE1, MAPT, and TNF. The results highlight the importance of these genes among the described data set to possess a role in Alzheimer’s management by *A. esculentus* seeds.

#### 3.3.4. Total Network Pharmacology

**Figure 4:**
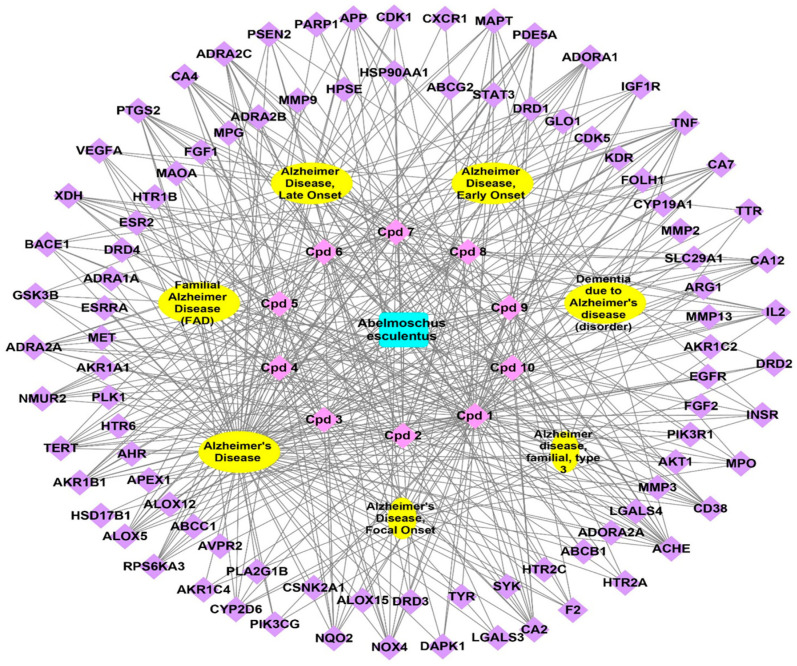
Complete pharmacology network (plant–compounds–genes–Alzheimer’s disorders); the network explains the relations between the plant, identified compounds, and target genes, in relation to Alzheimer’s disorders; the blue rectangle is the plant name, pink circles represent the identified compounds, yellow oval shapes represent Alzheimer’s disorders, and violet diamonds represent genes related to Alzheimer’s disorders.

Linking the previously constructed networks (plant–compounds, compounds–genes, and genes–Alzheimer’s disorders) in one network led to summarize the plant–compound–gene–Alzheimer’s interactions; the formed network consisted of 110 plant names, ten nodes for identified compounds, 92 nodes for genes related directly or indirectly to Alzheimer’s, and seven for Alzheimer’s, in addition to 444 edges representing interactions between nodes (Figure 4).

#### 3.3.5. Protein–Protein Interactions (PPI)

In the formed PPI network using the STRING database, non-interacting genes were not involved. The network was composed of 100 nodes and 145 edges with an average node degree of 2.9 (Figure S3A). The genes with the highest interactions among the PPI network were introduced as a subnetwork (Figure S3B); the PPI and the subnetwork identified PIK3R1, HSP90AA1, SRC, EGFR to be the highest-interacting genes in this gene set.

#### 3.3.6. Gene Ontology and Pathway Enrichment Analysis

All targets were introduced to the STRING database and the obtained results identified the target genes to affect biological processes, especially the cellular response to jasmonic acid stimulus (GO:0071395), the farnesol catabolic phase (GO:0016488), the daunorubicin metabolic process (GO:0044597), and the doxorubicin metabolic process (GO:0044598); the top-identified cellular components were the insulin receptor complex (GO:0005899), the phosphatidylinositol 3-kinase complex, class I (GO:0097651), the external side of the apical plasma membrane (GO:0098591), and glial cell projection (GO:0097386); top-identified molecular functions were phenanthrene 9,10-monooxygenase activity (GO:0018636), indanol dehydrogenase activity (GO:0047718), geranylgeranyl reductase activity (GO:0045550), and 17-alpha,20-alpha-dihydroxypregn-4-en-3-one dehydrogenase activity (GO:0047006); each class of terms were arranged in descending order according to strength (Table S9).

Using the KEGG Mapper pathway detection database implemented in the KEGG database, a diagrammatic illustration identified 43 biological pathways involved in Alzheimer’s disorders (hsa05010) as a mutation-caused aberrant PSEN1 to PERK-ATF4 signaling cascade; a mutation-caused aberrant Aβ to AGE-RAGE signaling pathway was among the top pathways identified (Figure S4).

Consequently, several genes related to the identified compounds were involved in all Alzheimer’s disorders, as a result of the current network pharmacology study. These genes were (ACHE, APP, BACE1, MAPT, and TNF), so these genes may be responsible for the anti-Alzheimer’s potential of *A. esculentus* seeds; previous studies concerning Alzheimer’s disorders therapy were acetylcholinesterase (AChE) inhibitors. These drugs reduce symptoms induced by the death of cholinergic neurons by inhibiting acetylcholine (ACh) turnover [56]. The brain produces abundant quantities of APP, a single-pass transmembrane protein. It is rapidly and intricately metabolized by a series of proteases related to Alzheimer’s disorder [57]. The all gene set were able to affect Alzheimer’s disorder through the mutation-caused aberrant PSEN1 to the PERK-ATF4 signaling pathway in addition to the mutation-caused aberrant Aβ to the AGE-RAGE signaling pathway, The PERK-Dependent Molecular Mechanisms was proven to have a role in the treatment of neurodegenerative disorders [58]. The gene set possesses effects on cellular response to jasmonic acid stimulus and farnesol catabolic process (biological processes), insulin receptor complex and phosphatidylinositol 3-kinase complex, class I (cellular components), phenanthrene 9,10-monooxygenase activity, and indanol dehydrogenase activity (molecular functions).

## 4. Materials and Methods

### 4.1. A. esculentus Material, Chemicals, Reagents, Extraction of A. esculentus Seeds

*A. esculentus* seeds were purchased from the market in Minia area, Egypt. The plant was identified by Prof. Abd El-Halim A. Mohammed (Horticultural Research Institute, Department of Flora and Phytotaxonomy Research, Dokki, Cairo, Egypt). Additionally, A voucher specimen (2022-BuPD 113) has been stored at the Department of Pharmacognosy, Faculty of Pharmacy, Beni-Suef University, Egypt. The solvents used during our work were purchased from El-Nasr Company for Pharmaceuticals and Chemicals (Giza, Egypt). Regarding the biological investigation, donepezil, all reagents and kits were obtained from Sigma Chemical Company (Saint Louis, MO, USA), while aluminum chloride (AlCl_3_) was obtained from CDH (Delhi, India).

Air-dried seeds (1.0 kg) were macerated in ethanol (70%, 1.5 L, 3×, 7 d each) kept at room temperature. The extract was additionally concentrated using reduced pressure to give a syrupy consistency utilizing a rotary evaporator (Buchi Rotavapor R-300, Cole-Parmer, Vernon Hills, IL, USA), kept at 45 °C to afford 20.0 g crude extract. Moreover, this was maintained at 4 °C for biological and metabolomic studies [38,59,60,61,62,63,64,65,66,67,68,69,70,71].

### 4.2. In Vitro DPPH Free Radical Scavenging Activity Assay of A. esculentus Seed Extract

The free radical scavenging potential of *A. esculentus* seed total extract was investigated using the DPPH assay (stable radical 2,2-diphenyl-1-picrylhydrazyl) [32]. In a brief manner at several concentrations (0.01, 0.05, and 0.1 μg mL^−1^ in absolute ethanol), 1 mL solution from our tested extract was added to 2 mL of DPPH solution (freshly prepared, 20 μg mL^−1^ in absolute ethanol). Moreover, the mixture was then kept at room temperature in a dark place for 30 min. Using a UV-Vis Jenway 6003 spectrophotometer, the absorbance was measured at *λ*_517_ (nm). Ascorbic acid in addition to absolute ethanol were utilized as a positive control as well as a blank, respectively. However, the following equation was used to calculate the DPPH radical scavenging activity:

$$\%\;DPPH\;scavenger\;activity=\frac{absorbance\;of\;blank-absorbance\;of\;tested\;sample}{absorbance\;of\;blank}\times 100$$

### 4.3. In Vitro Determination of Cholinesterase Potential of A. esculentus Seed Extract

The cholinesterase potential of *A. esculentus* seed total extract was assessed according to the manufacturer’s guidelines [72]. Accordingly, various concentrations of the tested extract (10 and 20 μg mL^−1^) in absolute ethanol were prepared. After that, 0.2 mL of these solutions were mixed with 3 mL distilled water followed by the addition of phosphate solution (3 mL), and the pH was determined with a pH meter (“pH-1”). Then, acetylcholine iodide solution (0.12 mL, 7.5%) was added and the mixture was kept at 37 °C in a water bath (for 30 min). Then, the pH value of these mixtures was recorded again (“pH-2”) and the difference between “pH-1” and “pH-2” within 30 min was calculated as a measure for the level of cholinesterase activity in the mixtures.

### 4.4. In Vivo Anti-Alzheimer’s Potential

#### 4.4.1. Animals

The animals used during this study were male rats (Wistar albino) (150 ± 10 g), obtained from the National Research Centre Animal House. The animals were divided into groups of ten rats for each cage, kept under environmental conditions well controlled at 26–29 °C. Additionally, they were supplied with a fixed light/dark cycle (1 week) as an interval to adapt under normal conditions, as they were permitted to have their water and food freely.

#### 4.4.2. Animal Ethical Report

Our study was authorized by the Ethical Committee of Beni-Suef University, Egypt, which demonstrated that the rats will not feel pain at any period of the experiments and be conserved in conformity with the instructions for the care and usage of laboratory animals (ethical approval no: 022-371).

#### 4.4.3. Induction of AlCl_3_-Induced Alzheimer’s Illness

AlCl_3_ solutions were prepared freshly at the onset of each experiment. Drinking water was used to dissolve AlCl_3_ to be administrated orally at a dose of 100 mg/kg to the animals every day for 8 weeks, 0.5 mL/100 g b.wt. [73].

#### 4.4.4. Acute Toxicity Investigation

The total extract was prepared at different concentrations for determining the acute toxicity, (from 500, 1000, 2000, 3000, and 4000, to 5000 mg/kg b.wt.), administered to four rats per each group (i.e., a total of 24 animals for all groups).

#### 4.4.5. Behavioral Measurement

Evaluation of the cognitive functions, as well as s motor coordination T-maze, were conducted at the National Research Centre (N.R.C.). After the chronic administration of AlCl_3_ (2 months), and at the end of treatment phase, the cognitive function and impairment of spatial memory of the animals were estimated [74]. Motor ability was evaluated using the beam balance investigation [75].

#### 4.4.6. Experimental Design, and Blood Samples Preparation

Rats used during this study were grouped randomly as six groups of ten animals each. Groups 1 and 2 served as normal, healthy control rats and AD rats, respectively, where AlCl_3_ was orally administrated to Group 2, while Groups 3 and 4 were the AD rats treated with *A. esculentus* seed’s total extract (500 mg/kg b.wt.) every day for 6 weeks (1/10 LD_50_) and the AD rats treated with the standard drug donepezil (10 mg/kg b.wt.) daily for 6 weeks [76].

Overnight fasted animals were sacrificed under slight thiopental anesthesia (30 mg/kg b.wt.) [77]. In a clean and dry test, the blood samples were gathered after anaesthetizing the rats utilizing a cardiac puncture. Blood samples were kept for 10 min until clotting and then centrifuged at 3000 rpm (1.175 g) to get serum. Additionally, the collected serum was kept at −80 °C for performing the biochemical investigations of IL-6, AChE, and TAC.

#### 4.4.7. Preparation of the Brain Tissue Samples

Upon completion of the experiment, the animals were fasted overnight and they were anesthetized and sacrificed [77]. Dissection of the entire brain of each animal was rapidly carried out, then the material was washed using isotonic saline and placed upon filter paper to dryness. Additionally, the collected brain from each rat was weighed as well as homogenized in ice-cold medium which contained 50 mM tris/HCl in addition to 300 mM sucrose at pH 7.4, giving a 10% (*w*/*v*) homogenate [73,78]. Centrifugation of the homogenate was carried out (1400× *g* for 10 min at 4 °C). Moreover, the supernatant was kept at −80 °C and screened against different biomarkers such as an antioxidant, oxidative stress, neurotransmitters, AGEs, and BDNF.

#### 4.4.8. Estimation of Brain Neurotransmitters

Serum acetylcholine esterase (AChE) was determined using a quantitative ELISA consistent with Engvall and Perlman, 1971 [79]. The concentrations of norepinephrine, dopamine, and serotonin in the brain were evaluated utilizing HPLC-ED consistent with Giday et al., 2009 [80]. IL-6, advance glycated end products, and BDNF concentrations were measured utilizing ELISA kits consistent with the manufacturer’s directions [80]. Moreover, TAC was evaluated in the serum according to Koracevic et al., 2009 [81]. In this method, 2,2′-azino-bis(3-ethylbenzthiazoline-6-sulfonic acid) (ABTS) was kept with met myoglobin as well as H_2_O_2_ to give the green radical cation ABTS+. Additionally, antioxidants make suppression for the color production by reducing the color intensity, which was relative to the concentration of the antioxidants and was evaluated at 600 nm using a microplate reading format. The animal groups were exposed to an evaluation for the non-enzymatic, reduced glutathione (GSH) (Beutler et al., 1963 [82]) and malondialdehyde (MDA) (Ohkaw et al., 1979 [83]). Additionally, the spectrophotometric/microplate reader analysis technique for glutathione (GSH) includes oxidation of GSH using the sulfhydryl reagent DTNB to obtain the yellow derivative TNB, which is measurable at 412 nm. Additionally, the principle of the MDA assay is built upon the measurement of the required absorbance at 535 nm as the spectrophotometrical measurement of the color which MDA forms with TBA in acidic media.

#### 4.4.9. Histopathological Investigations

The samples obtained from all rats’ brains per group were preserved in 10% neutral buffered formalin. Additionally, paraffin segments of 5 μm thickness were formulated and stained with hematoxylin in addition to eosin (H&E) for histopathological study, utilizing a light microscope (BX43, Olympus) and photographed using the Olympus software in connection with an Olympus DP27 camera [84]. An experienced pathologist carried out the histological analysis. Neuropathologic damage was evaluated on a scale from 0 to 4 as follows: (0) showed no changes; (1) revealed an area affected of <20%); (2) displayed an area affected of 20–30%); (3) showed an affected area of >30–60%); and (4) indicated an affected area of >60% [85].

### 4.5. Metabolomic Investigation Procedure

The crude extract from *A. esculentus* seeds was prepared at a concentration of 1 mg/mL for mass spectrometry investigation. The obtained ethanolic extract was investigated using metabolic study using LC-HR-ESI-MS consistent with Abdelmohsen et al., 2014 [86]. The acquired data from the investigated ethanolic extract were dereplicated using the DNP database [87,88].

### 4.6. Network Pharmacology Study

#### 4.6.1. Identified Compounds-Genes

The genes of each identified compound from *A. esculentus* seed extract were extracted from the chemistry database PubCHem (https://pubchem.ncbi.nlm.nih.gov/) [89] last accessed on (1 November 2022), and the Online SwissTARgetPrediction (http://www.swisstargetprediction.ch/) was also used [90] last accessed on (6 November 2022).

#### 4.6.2. Genes-Alzheimer’s Disorders

The genes linked to Alzheimer’s illness were collected from DisGeNet [91] last accessed on (10 November 2022). Additionally, the target genes UniProt IDs were chosen as input IDs in the DisGeNET database to find out which gene linked to Alzheimer’s disorders. In order to minimize the scope for the gene set to Alzheimer’s illness, the filter keywords of “Alzheimer’s disease”, we utilized “Alzheimer Disease, Early Onset”, “Alzheimer´s disease, focal onset”, “Alzheimer disease, late onset”, “Alzheimer disease, familial, type 3′”, “Familial Alzheimer’s Disease (FAD)”, and “Dementia due to Alzheimer’s disease (disorder)”.

#### 4.6.3. The Protein-Protein Interaction (PPI)

To investigate and display each potential interaction between the detected genes, the STRING database was utilized. (https://stringdb.org/cgi/network?taskId=bIDN4htc9NBY&sessionId=bZWvNlZHMn9h) [92] last accessed on (15 November 2022). The selected proteins were selected with the human species “*Homo sapiens*” as well as a confidence count higher than 0.4. STRING was utilized to discover proteins that interrelated with *A. esculentus* dereplicated metabolites specifying targets directly or indirectly to Alzheimer’s disorders’ subnetworks as constructed using the Cytoscape plugin CytoNCA application [91].

#### 4.6.4. Network Construction and Visualization

Plant–compounds, compounds–genes, PPI, and plant–compounds–genes– Alzheimer’s disorders networks were built using the Cytoscape network analysis program, version 3.9.0 [92]. The difference was significant at *p* < 0.05. Nodes stand for compounds, genes, and Alzheimer’s disease in the graphical network; additionally, edges stand for its related interactions.

#### 4.6.5. Gene Enrichment Investigation

The characterized pathways connected to *A. esculentus* related to Alzheimer’s disorders were retrieved by the STRING database (https://string-db.org/cgi/network?taskId=bIDN4htc9NBY&sessionId=bZWvNlZHMn9h) [92] last accessed on (15 November 2022) and the KEGG Mapper database (https://www.genome.jp/kegg/mapper/) last accessed on (20 November 2022) to consider the biological function, cellular elements, molecular functions, and incorporated biological pathways.

### 4.7. Statistical Investigation

All the obtained data were represented as mean ± SD. Additionally, the data were screened for normal distribution statistically utilizing one-way analysis of variance software as well as Co-State for Windows, version 8. The values of various letters are significant at *p* < 0.05 statistically.

$$\%\;change=\frac{mean\;of\;negative\;control-mean\;of\;treatment\;group}{mean\;of\;negative\;control}\times 100$$

$$\%\;improvement=\frac{mean\;of\;positive\;control-mean\;of\;treatment\;group}{mean\;of\;negative\;control}\times 100$$

## 5. Conclusions

In this study, *A. esculentus* seed crude extract showed significant neuroprotective, anti-apoptotic, and anti-amnesic potential against AlCl_3_-induced cerebral damages and cognitive dysfunctions, which could be associated with the antioxidant and the anti-AChE characters of the extract. Ten secondary metabolites were dereplicated using LC–HRESIMS. Network pharmacology analysis resulted in establishing a hypothesis indicating that the identified compounds from *A. esculentus* possess anti-Alzheimer’s effects through specific genes (ACHE, APP, BACE1, MAPT, and TNF). This study proposes the application of *A. esculentus* seed crude extract as a treasure remedy in AD treatment but more investigations, such as mechanistic investigations and quantification for the secondary metabolites in the extract, are necessary to validate the results.

## Figures and Tables

**Table 1:** In vitro DPPH scavenging activity of *A. esculentus* seed extract.

	0.01 µg/mL	0.05 µg/mL
Crude extract	77.2 ± 3.6 ^c^	93.2 ± 7.5 ^d^
Ascorbic acid	82.3 ± 4.7 ^b^	91.0 ± 6.1 ^d^

The data were expressed as mean ± SD (*n* = 3) of at least three independent experiments. Groups with similar letters are not significantly different, while those with different letters are significantly different at *p* ≤ 0.05.

**Table 2:** Effect of *A. esculentus* seed extract on AChE levels in Alzheimer’s-disorder-induced rats.

Parameters	Control	AlCl_3_-AD	Extract	Donepezil Drug
AChE (serum) (U\L) % Change % Improvement	215.0 ± 11.0 ^a^	419.0 ± 10.0 ^b^ +94.8	255.0 ± 8.0 ^c^ 76.2	240.0 ± 9.7 ^c^ 83.2

The data are represented as means ± SD (*n* = 10). Groups showing the same letters were not significantly different; additionally, those with different letters revealed considerable difference at *p* ≤ 0.05. AChE: acetylcholine esterase. The % change was calculated compared to the control group as (mean of treated—mean of negative/mean of negative control) × 100. The % improvement was also calculated as mean of positive control—mean of treated/mean of negative control × 100.

**Table 3:** Effects of *A. esculentus* seed extract on the levels of BDNF, glycated end product, and IL-6 in AD-induced rats.

	BDNF (pg/g Tissue)	Glycated End Product (μg/g Tissue)	IL-6 (pg/mL)
Control	343.0 ± 10.2 ^a^	12.7 ± 1.2 ^a^	39.6 ± 3.0 ^a^
AlCl_3_-AD % Change	167.0 ± 10.9 ^b^ −50.3%	40.8 ± 3.1 ^b^ 221.2%	130.0 ± 8.0 ^b^ 227.7%
AD crude extract % Improvement	258.0 ± 6.0 ^d^ 26.5	20.0 ± 2.0 ^d^ 163.7	45.3 ± 3.0 ^d^ 213.5
Donepezil drug % Improvement	288.0 ± 16.0 ^e^ 35.2	12.9 ± 1.1 ^a^ 219.6	39.0 ± 2.8 ^a^ 229.4

The data are shown in seconds as mean ± SD (*n* = 10). Groups showing the same letters are not significantly different; additionally, those with different letters revealed a significant difference at *p* ≤ 0.05. The % change was calculated compared to the control group as (mean of treated—mean of negative/mean of negative control) × 100. Additionally, the % improvement was calculated as (mean of positive control—mean of treated/mean of negative control) × 100.

## Data Availability

Not applicable.

## Supplementary Materials

The next supporting information are available to be downloaded at: https://www.mdpi.com/article/10.3390/plants12122382/s1. Table S1: In vitro AChE inhibition activity of *A. esculentus* seed extract, Table S2: Effect of *A. esculentus* seed extract using the T-maze test in Alzheimer’s disorders induced rats, Table S3: Effect of *A. esculentus* seed extract using beam balance test in Alzheimer’s disease induced rats, Table S4: Effect of *A. esculentus* seed extract on the levels of norepinephrine, dopamine and serotonin in Alzheimer’s disease induced rats, Table S5: Effects of *A. esculentus* seed extract on the levels of TAC, MDA, and GSH levels in AD-induced rats, Table S6: Scoring of histopathological alterations in brain of all treated groups, Table S7: Dereplicated metabolites from LC-HRESIMS analysis of *A. esculentus* seed extract, Table S8: Compounds-genes network analysis arranged according to number of undirected edges, Table S9: Gene ontology for top 15 terms related to the interacted genes among the data set of genes targeted by identified compounds of *A. esculentus*. Figure S1A: LC-HR-ESI-MS Chromatogram of the dereplicated metabolites of *A. esculentus* seed extract (positive), Figure S1B: LC-HR-ESI-MS Chromatogram of the dereplicated metabolites of *A. esculentus* seed extract (negative), Figure S2: Genes-Alzheimer network; the network shows the genes in the described data set related to different typed of Alzheimer, green triangles represent genes related to Alzheimer, yellow oval shapes represent types of Alzheimer’s disease, Figure S3: PPI network of interacted genes of dataset of genes targeted by identified compounds of *A. esculentus*, A: total interacted PPI, B: subnetwork of top interacted genes, Figure S4: KEGGMAPPER diagram for the biological pathways related to Alzheimer’s diseases through all gene set identified.
