# Supplementary material for: Abelmoschus eculentus Seed Extract Exhibits In Vitro and In Vivo Anti-Alzheimer’s Potential Supported by Metabolomic and Computational Investigation
Source: Plants (Basel). 2023 Jun 20;12(12):2382. doi: 10.3390/plants12122382 (PMC10304855; doi:10.3390/plants12122382)
Supplement: Supplementary file 1 [file plants-12-02382-s001.zip › plants-2403434-supplementary.pdf]

Abelmoschus esculentus **Seed Extract Exhibits** ***In Vitro*** **and** ***In Vivo*** **Anti-Alzheimer Potential Supported by Metabolomic and Computational Investigation**

Hussain T. Bakhsh[1≠], Fatma A. Mokhtar[2≠], Abeer H. Elmaidomy[3], Hanan F. Aly[4], Eman A. Younis[4], Mubarak A. Alzubaidi[5], Faisal H. Altemani[6], Naseh A. Algehainy[6], Mohammed Majrashi[7], Faisal Alsenani[8], Gerhard Bringmann[9*], Usama Ramadan Abdelmohsen[10,11*], Omnia Hesham Abdelhafez[11*]

[1]Department of Pharmacy Practice, Faculty of Pharmacy, King Abdulaziz University, Jeddah 21589, Saudi Arabia.

[2]Department of Pharmacognosy, Faculty of Pharmacy, El Saleheya El Gadida University, El Saleheya El Gadida, Sharkia, Egypt.

[3]Department of Pharmacognosy, Faculty of Pharmacy, Beni-Suef University, Beni-Suef 62514, Egypt.

[4]Department of Therapeutic Chemistry, National Research Centre (NRC), El-Bouth St., P.O. 12622 Cairo, Egypt.

[5]Department of Biological Sciences, Faculty of Science, King Abdulaziz University, Jeddah 21589, Saudi Arabia.

[6]Department of Medical Laboratory Technology, Faculty of Applied Medical Sciences, University of Tabuk, Tabuk 71491.

[7]Department of Pharmacology, Faculty of Medicine, University of Jeddah, Jeddah 23881, Saudi Arabia.

[8]Department of Pharmacognosy, College of Pharmacy, Umm Al-Qura University, Makkah 21955, Saudi Arabia.

[9]Institute of Organic Chemistry, University of Würzburg, Am Hubland, 97074 Würzburg, Germany.

[10]Department of Pharmacognosy, Faculty of Pharmacy, Minia University, Minia 61519, Egypt.

[11]Department of Pharmacognosy, Faculty of Pharmacy, Deraya University, 7 Universities Zone, New Minia 61111, Egypt.

[≠] These authors contributed equally.

[*]Authors to whom correspondence should be addressed:

bringmann@chemie.uni-wuerzburg.de, usama.ramadan@mu.edu.eg, omnia.hesham@deraya.edu.eg

Table S1. In vitro AChE inhibition activity of Abelmoschus esculentus seed extract.

	IC_{50} µM
Crude extract	0.028 ± 0.003[a]
Donepezil	0.025 ± 0.003[a]

The data were expressed as mean ±SD (n = 3) of at least three independent experiments. The differences among various treatment groups were determined by One Way Analysis of Variance (ANOVA) software and Co–State for Windows, version 8). Donepezil was used as a positive control. IC_{50} = the concentration (µM) that caused a 50% inhibition of cell growth *in vitro*.

Table S2. Effect of Abelmoschus esculentus seed extract using the T- maze test in Alzheimer's disorders induced rats.

	Baseline	Induction (2 months)	Treatment (6 weeks)
Control	14.3 ± 1.2[a]	15.8 ± 1.2[a]	14.9 ± 0.9[a]
$AlCl_3$ -AD	16.3 ± 1.1[a]	52.9 ± 3.6[b]	-
% Change	+13.8	+233.8	
Crude extract	-	-	25.1 ± 1.0[d]
% Improvement			175.5
Donepezil drug	-	-	24.1 ± 0.7[d]
% Improvement			181.9

The data were expressed in seconds as mean ±SD (n = 10). Groups with similar letters are not significantly different, while those with different letters are significantly different at $p \leq 0.05$. The % change is calculated compared to control group as: (mean of treated − mean of negative / mean of negative control) × 100. The % improvement is calculated: mean of positive control – mean of treated / mean of negative control × 100.

Table S3. Effect of Abelmoschus esculentus seed extract using beam balance test in Alzheimer's disease induced rats.

	Baseline	Induction (2 months)	Treatment (6 weeks)
Control	10.6 ± 0.8[a]	10.1 ± 0.4[a]	11.1 ± 0.2[a]
$AlCl_3$-AD	10.0 ± 1.0[a]	2.9 ± 0.6[b]	-
% Change		70.6	
Crude extract	-	-	9.2 ± 1.1[d]
% Improvement			62.0
Donepezil drug	-	-	10.0 ± 0.5[d]
% Improvement			69.0

The data were expressed in seconds as mean ±SD (n = 10). Groups with similar letter are not significantly different, while those with different letter are significantly different at $p \leq 0.05$. The % change is calculated compared to control group as: (mean of treated − mean of negative / mean of negative control) × 100. The % improvement is calculated: mean of positive control – mean of treated / mean of negative control × 100.

Table S4. Effect of Abelmoschus esculentus seed extract on the levels of norepinephrine, dopamine and serotonin in Alzheimer's disease induced rats.

	Norepinephrine (µg/g tissue)	Dopamine (µg/g tissue)	Serotonin (5-HT) (µg/g tissue)
Control	2.0 ± 0.0[a]	3.2 ± 0.2[a]	2.9 ± 0.2[a]
$AlCl_3$-AD	16.4 ± 0.9[b]	1.0 ± 0.0 [b]	1.0 ± 0.0[b]
% Change	+723.00	-68.9	-65.6
Crude extract	5.5 ± 0.3[a]	2.4 ± 0.6[c]	2.5 ± 0.1[c]
% Improvement	545.0	46.2	50.8
Donepezil drug	5.3 ± 0.5[c]	2.7 ± 0.5[c]	2.6 ± 0.1[c]
% Improvement	558.0	55.2	54.8

The data were expressed as means ± SD (n = 10). Groups with similar letters are not significantly different, while those with different letters are significantly different at $p \leq 0.05$. The % change is calculated compared to control group as: (mean of treated − mean of negative / mean of negative control) × 100. The % improvement is calculated: mean of positive control – mean of treated / mean of negative control × 100.

Table S5. Effects of Abelmoschus esculentus seed extract on the levels of TAC, MDA, and GSH levels in AD-induced rats.

Groups	Control rats	Intoxicated rats	Crude extract	Standard Drug
Parameters				
TAC (mM/L)	0.8 ± 0.3[a]	0.2 ± 0.2[d]	0.6 ± 0.1[b]	0.7 ± 0.2[b]
% Change	-	-74.1		
% Improvement			51.6	52.8
MDA (nmol/g tissue)	26.1 ± 2.0[d]	144.0 ± 6.1[a]	42.1 ± 3.2[b]	41.1 ± 2.5[b]
% Change	-	+451.7		
% Improvement			390.4	394.2
GSH (tissue) (mg/g tissue)	25.0 ± 1.0[a]	9.7 ± 3[d]	21.0 ± 1.0[b]	23.1 ± 2.2[b]
% Change		60.9		
% Improvement			44.9	53.4

The data are expressed in seconds as mean ± SD (n = 10). Groups with similar letters are not significantly different, while those with different letters are significantly different at $p \leq 0.05$. The % change is calculated compared to control group as: (mean of treated − mean of negative / mean of negative control) × 100. The % improvement is calculated: mean of positive control – mean of treated / mean of negative control × 100.

Table S6. Scoring of histopathological alterations in brain of all treated groups.

Lesions	G1 Control	G2 Alzheimer	G3 crude extract	G4 Drug
Neuronal degeneration with NFT	0	3	1	1
Congestion of meningeal blood vessels	0	2	0	0
Meningeal hemorrhage	0	2	1	1
Degeneration of hippocampus neurons	0	3	1	1
Degeneration of *Purkinje* cells in cerebellum	0	2	0	0
Decreased granular layer density in cerebellum	0	2	0	0

The score system was designed as: score 0 = absence of the lesion in all rats of the group (n = 5), score 1 (< 30%), score 2 (< 30% – 50%), score 3 (> 50%). G1: control group, G2 Alzheimer induced group, G3 *Abelmoschus esculentus* seed extract–treated AD group, and G4 donepezil-treated AD group.

Table S7. Dereplicated metabolites from LC-HRESIMS analysis ofAbelmoschus esculentus seed extract.

Nu.	Metabolite name	Original source	MF	RT (min)	*m/z*
**1**	Quercetin	*Abelmoschus manihot*	$C_{15}H_{10}O_7$	3.0908222	301.0353-
**2**	4-*O*-*α*-D-Galactopyranosyl-D-galactose	*Hibiscus esculentus*	$C_{12}H_{22}O_{11}$	1.3379	341.10792-
**3**	Isoquercitrin	*Abelmoschus esculentus*	$C_{21}H_{20}O_{12}$	2.9908210	465.1033+
**4**	Quercetin 3-glycosides; 3-*O*-(4-*O*-Methyl-*β*-D-glucopyranoside)	*Abelmoschus esculentus*	$C_{22}H_{22}O_{12}$	2.8314813	477.10499-
**5**	Tiliroside	*Abelmoschus manihot*	$C_{30}H_{26}O_{13}$	2.4316013	595.1452+
**6**	5,7,3′,4′-Tetrahydroxy flavonol-3-*O*-[*β*-d-rhamnopyranosil-(1→2)]-*β*-d-glucopyranoside	*Abelmoschus esculentus*	$C_{26}H_{28}O_{26}$	2.7412710	597.1456+
**7**	Quercetin-3-Orobinoside	*Abelmoschus manihot*	$C_{27}H_{30}O_{16}$	2.2112778	609.1466-
**8**	Floramanoside D	*Abelmoschus manihot*	$C_{28}H_{32}O_{16}$	2.2232778	625.1769+
**9**	Quercetin-3-*O*-sophoroside	*Abelmoschus esculentus*	$C_{27}H_{30}O_{17}$	2.3412578	627.1561+
**10**	3-*O*-Kaempferol-2-O-acetyl-4-*O*-(*p*-coumaroyl)-*α*-D-glucopyranoside	*Abelmoschus manihot*	$C_{32}H_{28}O_{14}$	2.1514013	637.1557+

MF: molecular formula, RT: retention time, min: minute, *m/z*: mass-to-charge ratio

Table S8: Compounds-genes network analysis arranged according to number of undirected edges

Node name	Number of undirected edges	Degree	Betweenness centrality	Topological coefficient	Average shortest path length
**Quercetin**	100	100	0.841502538	0.107744108	1.572413793
**4-O-*α*-D-Galactopyranosyl-D-galactose**	31	31	0.339722802	0.093189964	2.510344828
**Isoquercitrin**	22	22	0.025241444	0.803030303	2.634482759
**Quercetin 3-glycosides; 3-O-(4-O-Methyl-*β*-D-glucopyranoside)**	22	22	0.025241444	0.803030303	2.634482759
**5,7,3′,4′-tetrahydroxy flavonol-3-*O*-[*β*-d-rhamnopyranosil-(1→2)]-*β*-d-glucopyranoside**	21	21	0.018392785	0.835978836	2.648275862
**Quercetin-3-Orobinoside**	21	21	0.018392785	0.835978836	2.648275862
**Floramanoside D**	21	21	0.018392785	0.835978836	2.648275862
**Quercetin-3-O-sophoroside**	21	21	0.018392785	0.835978836	2.648275862
**3-*O*-kaempferol-2-*O*-acetyl-4-*O*-(*p*-coumaroyl)-*α*-D-glucopyranoside**	21	21	0.018392785	0.835978836	2.648275862
**Tiliroside**	17	17	0.012247385	0.862745098	2.703448276
**P00918**	10	10	0.154472173	0.211851852	1.931034483
**Q9NPH5**	9	9	0.01300307	0.263374486	2.317241379
**P15121**	9	9	0.01300307	0.263374486	2.317241379
**P47989**	9	9	0.01300307	0.263374486	2.317241379
**P30542**	9	9	0.01300307	0.263374486	2.317241379
**P43166**	9	9	0.01300307	0.263374486	2.317241379
**O43570**	9	9	0.01300307	0.263374486	2.317241379
**P22748**	9	9	0.01300307	0.263374486	2.317241379
**P22303**	9	9	0.01300307	0.263374486	2.317241379
**P28907**	9	9	0.01300307	0.263374486	2.317241379
**P08913**	9	9	0.02365235	0.426303855	3.131034483
**P18825**	9	9	0.02365235	0.426303855	3.131034483
**P09917**	8	8	0.011473211	0.277777778	2.331034483
**O14746**	8	8	0.011473211	0.277777778	2.331034483
**P16083**	8	8	1.85E-04	0.94047619	3.531034483

**P51812**	8	8	1.85E-04	0.94047619	3.531034483
**Q9GZQ4**	8	8	1.85E-04	0.94047619	3.531034483
**P35354**	8	8	1.85E-04	0.94047619	3.531034483
**O76074**	8	8	1.85E-04	0.94047619	3.531034483
**P01375**	7	7	1.18E-04	0.965986395	3.544827586
**P60568**	7	7	1.18E-04	0.965986395	3.544827586
**P06493**	3	3	0.118001876	0.503937008	2.151724138
**Q99808**	2	2	4.35E-06	1	3.613793103
**P30518**	1	1	0	0	2.565517241
**P21397**	1	1	0	0	2.565517241
**P08069**	1	1	0	0	2.565517241
**P36888**	1	1	0	0	2.565517241
**P11511**	1	1	0	0	2.565517241
**P00533**	1	1	0	0	2.565517241
**P00734**	1	1	0	0	2.565517241
**P11309**	1	1	0	0	2.565517241
**Q96GD4**	1	1	0	0	2.565517241
**P21917**	1	1	0	0	2.565517241
**Q04760**	1	1	0	0	2.565517241
**P05164**	1	1	0	0	2.565517241
**P27986**	1	1	0	0	2.565517241
**P29274**	1	1	0	0	2.565517241
**P53355**	1	1	0	0	2.565517241
**P06737**	1	1	0	0	2.565517241
**P00915**	1	1	0	0	2.565517241
**P49841**	1	1	0	0	2.565517241
**P12931**	1	1	0	0	2.565517241
**Q05397**	1	1	0	0	2.565517241
**P37059**	1	1	0	0	2.565517241
**P35968**	1	1	0	0	2.565517241
**P45452**	1	1	0	0	2.565517241
**P08254**	1	1	0	0	2.565517241
**P07451**	1	1	0	0	2.565517241
**P16050**	1	1	0	0	2.565517241
**P33527**	1	1	0	0	2.565517241
**P53350**	1	1	0	0	2.565517241
**P23280**	1	1	0	0	2.565517241
**P14780**	1	1	0	0	2.565517241
**P08253**	1	1	0	0	2.565517241
**Q16512**	1	1	0	0	2.565517241
**Q9ULX7**	1	1	0	0	2.565517241
**Q16790**	1	1	0	0	2.565517241
**P68400**	1	1	0	0	2.565517241
**P18054**	1	1	0	0	2.565517241
**P08581**	1	1	0	0	2.565517241
**P51955**	1	1	0	0	2.565517241

**P25024**	1	1	0	0	2.565517241
**Q13554**	1	1	0	0	2.565517241
**Q9UM73**	1	1	0	0	2.565517241
**P31749**	1	1	0	0	2.565517241
**P08183**	1	1	0	0	2.565517241
**Q9HC98**	1	1	0	0	2.565517241
**P04054**	1	1	0	0	2.565517241
**P35218**	1	1	0	0	2.565517241
**P56817**	1	1	0	0	2.565517241
**Q16678**	1	1	0	0	2.565517241
**P30530**	1	1	0	0	2.565517241
**Q9UNQ0**	1	1	0	0	2.565517241
**O60285**	1	1	0	0	2.565517241
**P52895**	1	1	0	0	2.565517241
**Q04828**	1	1	0	0	2.565517241
**P42330**	1	1	0	0	2.565517241
**P17516**	1	1	0	0	2.565517241
**Q8N1Q1**	1	1	0	0	2.565517241
**P14550**	1	1	0	0	2.565517241
**Q9HC97**	1	1	0	0	2.565517241
**P10636**	1	1	0	0	2.565517241
**B2RXH2**	1	1	0	0	2.565517241
**P11388**	1	1	0	0	2.565517241
**P06213**	1	1	0	0	2.565517241
**Q15746**	1	1	0	0	2.565517241
**P43405**	1	1	0	0	2.565517241
**P48736**	1	1	0	0	2.565517241
**P27695**	1	1	0	0	2.565517241
**Q13332**	1	1	0	0	2.565517241
**Q92731**	1	1	0	0	2.565517241
**P29372**	1	1	0	0	2.565517241
**Q96S37**	1	1	0	0	2.565517241
**Q15078**	1	1	0	0	2.565517241
**P05089**	1	1	0	0	2.565517241
**Q00534**	1	1	0	0	2.565517241
**P24941**	1	1	0	0	2.565517241
**P14679**	1	1	0	0	2.565517241
**P14061**	1	1	0	0	2.565517241
**P35869**	1	1	0	0	2.565517241
**P11474**	1	1	0	0	2.565517241
**P05067**	1	1	0	0	2.565517241
**P09874**	1	1	0	0	2.565517241
**P02766**	1	1	0	0	2.565517241
**P39900**	1	1	0	0	2.565517241
**O60218**	1	1	0	0	2.565517241
**Q9H2K2**	1	1	0	0	2.565517241

**O95271**	1	1	0	0	2.565517241
**P11387**	1	1	0	0	2.565517241
**P15692**	1	1	0	0	3.503448276
**P05230**	1	1	0	0	3.503448276
**Q9Y251**	1	1	0	0	3.503448276
**P07900**	1	1	0	0	3.503448276
**P49810**	1	1	0	0	3.503448276
**P09038**	1	1	0	0	3.503448276
**P56470**	1	1	0	0	3.503448276
**P17931**	1	1	0	0	3.503448276
**O00214**	1	1	0	0	3.503448276
**P41595**	1	1	0	0	3.503448276
**P18089**	1	1	0	0	3.503448276
**P21728**	1	1	0	0	3.503448276
**P14416**	1	1	0	0	3.503448276
**P25100**	1	1	0	0	3.503448276
**P28223**	1	1	0	0	3.503448276
**P28335**	1	1	0	0	3.503448276
**P35462**	1	1	0	0	3.503448276
**P10635**	1	1	0	0	3.503448276
**P50406**	1	1	0	0	3.503448276
**P35348**	1	1	0	0	3.503448276
**P28222**	1	1	0	0	3.503448276
**P51449**	1	1	0	0	3.503448276
**P40763**	1	1	0	0	3.503448276
**Q04609**	1	1	0	0	3.503448276
**Q9UQ49**	1	1	0	0	3.503448276
**Q9Y3R4**	1	1	0	0	3.503448276
**Q8WWR8**	1	1	0	0	3.503448276

Table S9: *Gene ontology for top 15 terms related to the interacted genes among the data set of genes targeted by identified compounds of Abelmoschus esculentus.*

**Term ID (GO term)**	**Term description**	**Strength**
**biological processes**		
**GO:0071395**	Cellular response to jasmonic acid stimulus	2.29
**GO:0016488**	Farnesol catabolic process	2.29
**GO:0044597**	Daunorubicin metabolic process	2.18
**GO:0044598**	Doxorubicin metabolic process	2.18
**GO:2001303**	Lipoxin a4 biosynthetic process	2.12
**GO:0032431**	Activation of phospholipase a2 activity	1.99
**GO:0035814**	Negative regulation of renal sodium excretion	1.99
**GO:0040016**	Embryonic cleavage	1.89
**GO:0061370**	Testosterone biosynthetic process	1.89
**GO:0071799**	Cellular response to prostaglandin d stimulus	1.89
**GO:1990962**	Xenobiotic transport across blood-brain barrier	1.89
**GO:0070212**	Protein poly-adp-ribosylation	1.87
**GO:0007077**	Mitotic nuclear envelope disassembly	1.81

**GO:0006689**	Ganglioside catabolic process	1.81
**GO:1901509**	Regulation of endothelial tube morphogenesis	1.81
**molecular functions**		
**GO:0018636**	Phenanthrene 9,10-monooxygenase activity	2.29
**GO:0047718**	Indanol dehydrogenase activity	2.29
**GO:0045550**	Geranylgeranyl reductase activity	2.29
**GO:0047006**	17-alpha,20-alpha-dihydroxypregn-4-en-3-one dehydrogenase activity	2.29
**GO:0047977**	Hepoxilin-epoxide hydrolase activity	2.29
**GO:0047086**	Ketosteroid monooxygenase activity	2.19
**GO:0047115**	trans-1,2-dihydrobenzene-1,2-diol dehydrogenase activity	2.17
**GO:0047655**	Allyl-alcohol dehydrogenase activity	2.17
**GO:0004052**	Arachidonate 12(s)-lipoxygenase activity	2.12
**GO:0005009**	Insulin-activated receptor activity	2.12
**GO:0016165**	Linoleate 13s-lipoxygenase activity	2.12
**GO:0050473**	Arachidonate 15-lipoxygenase activity	2.12
**GO:0051425**	PTB domain binding	2.12
**GO:0032052**	Bile acid binding	2.07
**GO:0004032**	alditol:NADP+ 1-oxidoreductase activity	2.06
**cellular components**		
**GO:0005899**	Insulin receptor complex	1.99
**GO:0097651**	Phosphatidylinositol 3-kinase complex, class i	1.89
**GO:0098591**	External side of apical plasma membrane	1.89
**GO:0097386**	Glial cell projection	1.24
**GO:0051233**	Spindle midzone	1.21
**GO:0000784**	Nuclear chromosome, telomeric region	1.06
**GO:1902911**	Protein kinase complex	1.01
**GO:0099056**	Integral component of presynaptic membrane	1.01
**GO:0061695**	Transferase complex, transferring phosphorus-containing groups	0.82
**GO:0099699**	Integral component of synaptic membrane	0.79
**GO:0000922**	Spindle pole	0.78
**GO:0033643**	Host cell part	0.76
**GO:0098687**	Chromosomal region	0.74
**GO:0060205**	Cytoplasmic vesicle lumen	0.73
**GO:0034774**	Secretory granule lumen	0.68

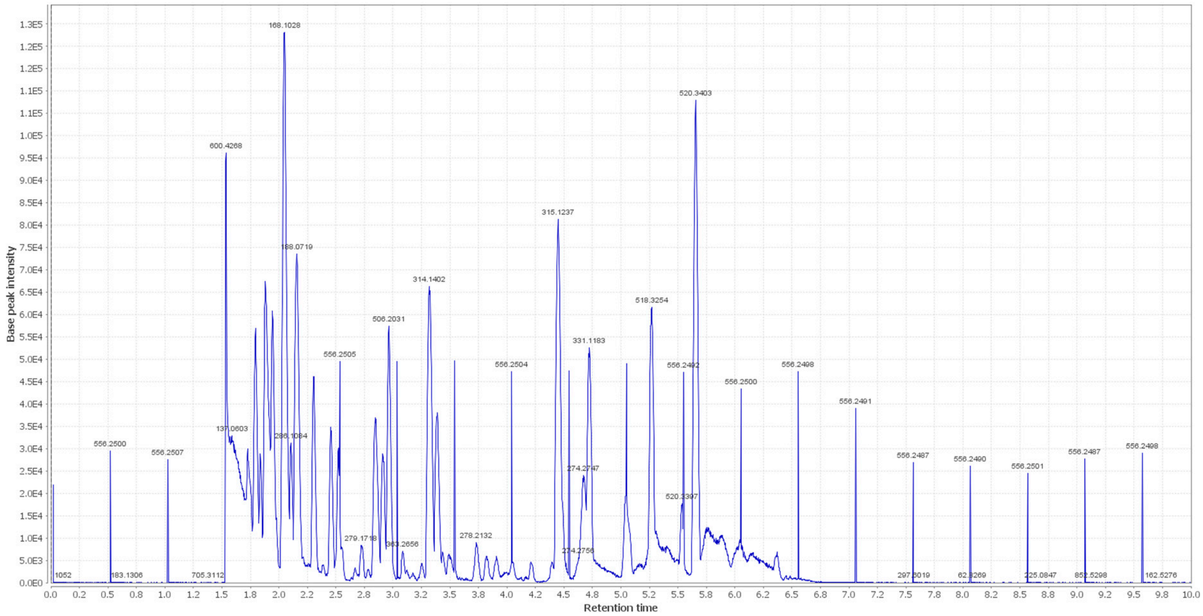

Figure S1A. LC-HR-ESI-MS Chromatogram of the dereplicated metabolites of *Abelmoschus esculentus* seed extract (positive).

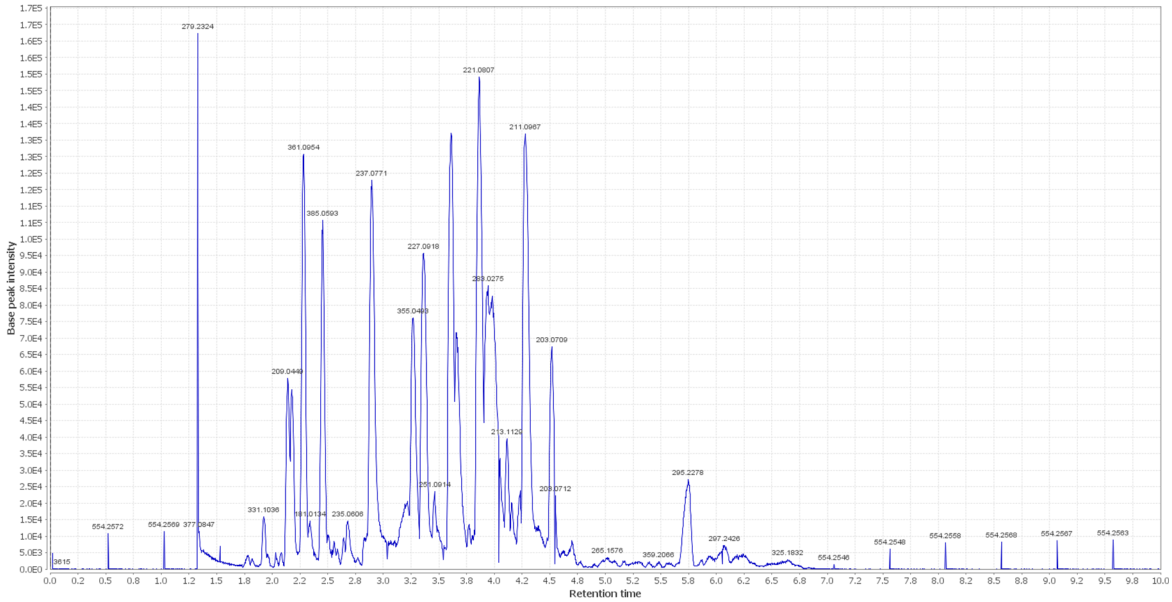

Figure S1B. LC-HR-ESI-MS Chromatogram of the dereplicated metabolites of *Abelmoschus esculentus* seed extract (negative).

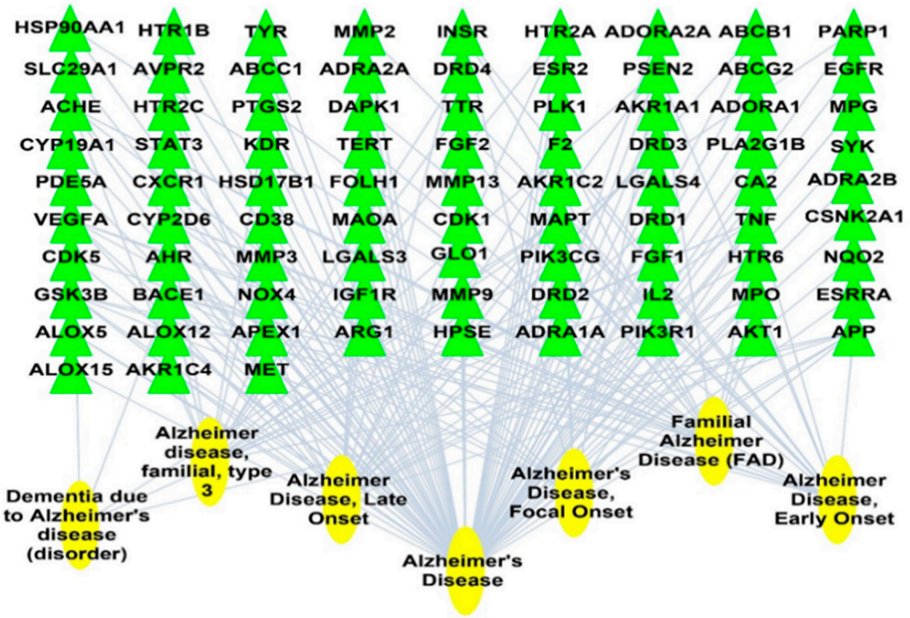

Figure S2: Genes-Alzheimer network; the network shows the genes in the described data set related to different typed of Alzheimer, green triangles represent genes related to Alzheimer, yellow oval shapes represent types of Alzheimer's disease.

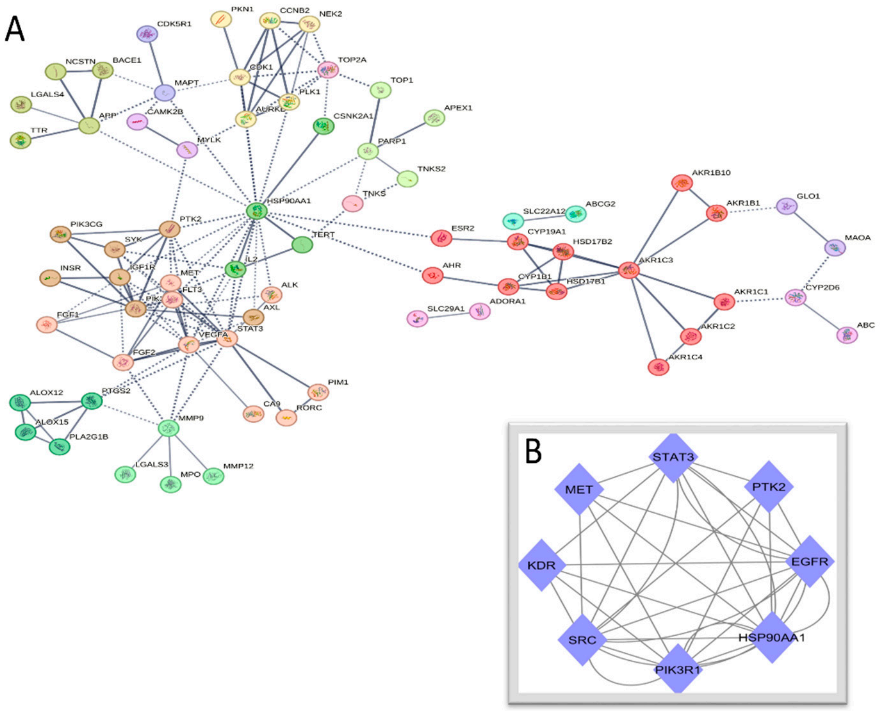

Figure S3: PPI network of interacted genes of dataset of genes targeted by identified compounds of *Abelmoschus esculentus*, A: total interacted PPI, B: subnetwork of top interacted genes.

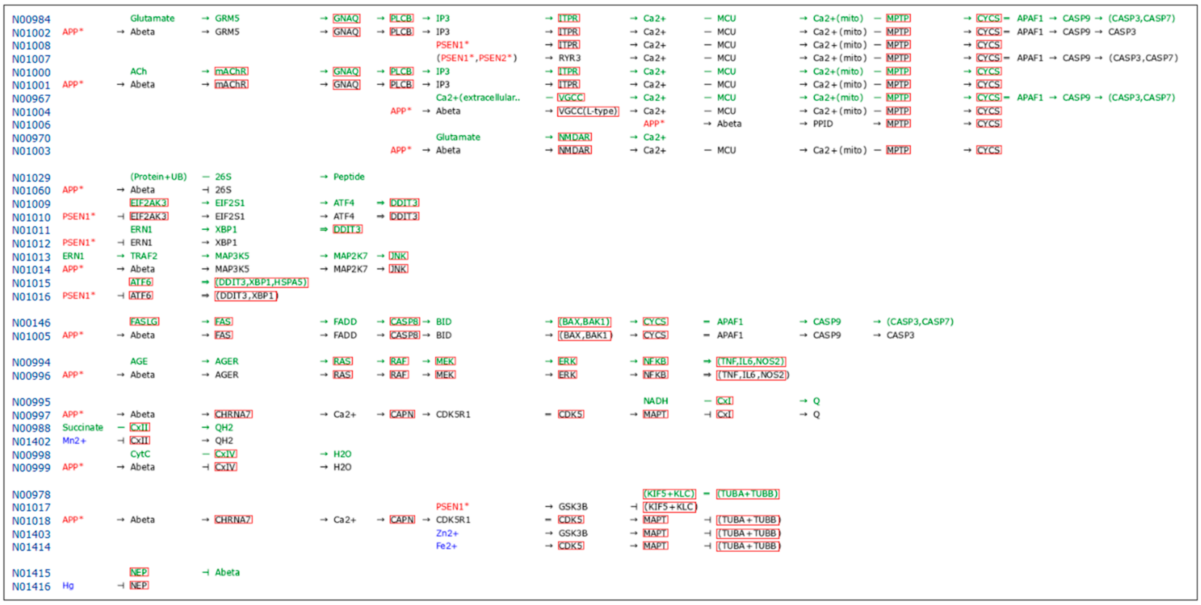

Figure S4: KEGGMAPPER diagram for the biological pathways related to Alzheimer's diseases through all gene set identified.
